# Nucleocytoplasmic Communication in Healthy and Diseased Plant Tissues

**DOI:** 10.3389/fpls.2021.719453

**Published:** 2021-07-28

**Authors:** Daniel Lüdke, Philipp F. W. Rohmann, Marcel Wiermer

**Affiliations:** ^1^Molecular Biology of Plant-Microbe Interactions Research Group, Albrecht-von-Haller-Institute for Plant Sciences, University of Göttingen, Göttingen, Germany; ^2^Molecular Biology of Plant-Microbe Interactions Research Group, Göttingen Center for Molecular Biosciences, University of Göttingen, Göttingen, Germany

**Keywords:** nuclear pore complex (NPC), nucleoporins (NUPs), nuclear transport receptors (NTRs), nucleocytoplasmic transport, plant development and immunity

## Abstract

The double membrane of the nuclear envelope (NE) constitutes a selective compartment barrier that separates nuclear from cytoplasmic processes. Plant viability and responses to a changing environment depend on the spatial communication between both compartments. This communication is based on the bidirectional exchange of proteins and RNAs and is regulated by a sophisticated transport machinery. Macromolecular traffic across the NE depends on nuclear transport receptors (NTRs) that mediate nuclear import (i.e. importins) or export (i.e. exportins), as well as on nuclear pore complexes (NPCs) that are composed of nucleoporin proteins (NUPs) and span the NE. In this review, we provide an overview of plant NPC- and NTR-directed cargo transport and we consider transport independent functions of NPCs and NE-associated proteins in regulating plant developmental processes and responses to environmental stresses.

## Nuclear Pore Complexes – Selective Transport Hubs for Macromolecular Exchange Between the Cytoplasm and the Nucleus

The nuclear envelope (NE) is a key compartment border of eukaryotic cells, partitioning cytoplasmic and nuclear processes. It consists of an outer nuclear membrane (ONM) and an inner nuclear membrane (INM). These two lipid bilayers enclose an intermembrane lumen that is termed the perinuclear space and is continuous with the lumen of the endoplasmic reticulum (ER). The partitioning of essential cellular processes by the NE, such as nuclear transcription of genetic information and the cytoplasmic translation of transcripts into proteins by ribosomes, requires the regulated exchange of molecular information between both compartments. The primary pathways for the bidirectional communication across the NE are nuclear pore complexes (NPCs). NPCs are supramolecular protein conglomerates that consist of multiple copies of approximately 30 nucleoporins (NUPs) and fuse the ONM and INM to form a central transport channel across the NE (Tamura and Hara-Nishimura, 2013; Schwartz, 2016; Tang et al., 2020). Nucleoporins containing intrinsically disordered phenylalanine (F)-glycine (G) repeat domains form a selective permeability barrier within the central channel. This barrier prevents the passive diffusion of soluble molecules > ∼40 kDa but also enables the energy-dependent selective translocation of nuclear transport receptors (NTRs) that bind localization motifs of macromolecular cargos (Christie et al., 2016; Raveh et al., 2016; Schmidt and Görlich, 2016). The small GTPase RAS-RELATED NUCLEAR PROTEIN (RAN) in its GTP-bound nuclear and GDP-bound cytoplasmic states both energizes and determines the directionality of nucleocytoplasmic transport mediated by NTRs of the karyopherin family (Nielsen, 2020). Inside the nucleoplasm, Ran⋅GTP dissociates imported cargos from nuclear import receptors (termed importins), but stabilizes the association of export receptors (termed exportins) with their nuclear cargo substrates. After translocation into the cytoplasm, exportin/cargo complexes dissociate due to the hydrolysis of Ran⋅GTP to Ran⋅GDP by RAN GTPase-ACTIVATING PROTEIN (RanGAP) and its co-factor RAN BINDING PROTEIN (RanBP; Nielsen, 2020).

Macromolecules that are transported in a Ran⋅GTP/GDP gradient-dependent manner *via* NTRs of the karyopherin family include proteins as well as different RNA species that are generated inside the nucleus, such as transfer-RNAs (tRNAs) and diverse regulatory small RNAs and ribosomal RNAs (rRNAs; Köhler and Hurt, 2007). By contrast, the export of messenger RNAs (mRNAs) operates independently of the RAN cycle and involves a conserved export receptor heterodimer (termed Mex67-Mtr2 in yeast and TAP-p15 in metazoans) that is structurally unrelated to karyopherins. This general mRNA export receptor operates together with RNA-binding proteins (RBPs) and processing factors that are recruited to the mRNA during messenger ribonucleoprotein particle (mRNP) biogenesis and nuclear export (Segref et al., 1997; Grüter et al., 1998; Sträßer et al., 2002; Masuda et al., 2005; Katahira, 2012; Ehrnsberger et al., 2019). The unidirectionality of mRNA transport through the NPC is imposed by an mRNP remodeling machinery on the cytoplasmic side of the NPC that frees the mRNA for ribosomal translation in the cytoplasm (Snay-Hodge et al., 1998; Tseng et al., 1998; Stewart, 2010; Ehrnsberger et al., 2019). It should be noted, that our current understanding of nuclear transport principles and the NPC structure is largely derived from work in yeast and vertebrates, and divergence from the described transport mechanisms may exist in different eukaryotes, including plants. This also includes trypanosomes that appear to employ a RAN-dependent system for mRNA export similar to protein transport (Obado et al., 2016).

Besides their fundamental functions in controlling the selective bidirectional exchange of macromolecules between the nucleoplasm and the cytoplasm, NPCs also play transport independent roles in several other cellular processes, including the spatial chromatin organization and gene positioning at the nuclear periphery to regulate gene expression in response to developmental and environmental stimuli (Meier et al., 2017; Groves et al., 2020).

## NPC and NTR Functions in Plant Development and Environmental Responses

Plants have to integrate information on a multitude of abiotic and biotic environmental stimuli with endogenous developmental programs to ensure proper growth and reproduction. Adaptations to these stimuli requires the dynamic signal transmission across the NE to drive changes in gene expression upon response pathway activation by cell surface or intracellular receptors. Consistent with nucleoporins being the building blocks of the multifunctional NPC, and NTRs mediating cargo translocation across the NPC, several nucleoporins and NTRs have been shown to participate in different molecular processes during plant adaptations to developmental and environmental cues.

### NTR Functions in Plant Development and Stress Signaling

**FIGURE 1:**
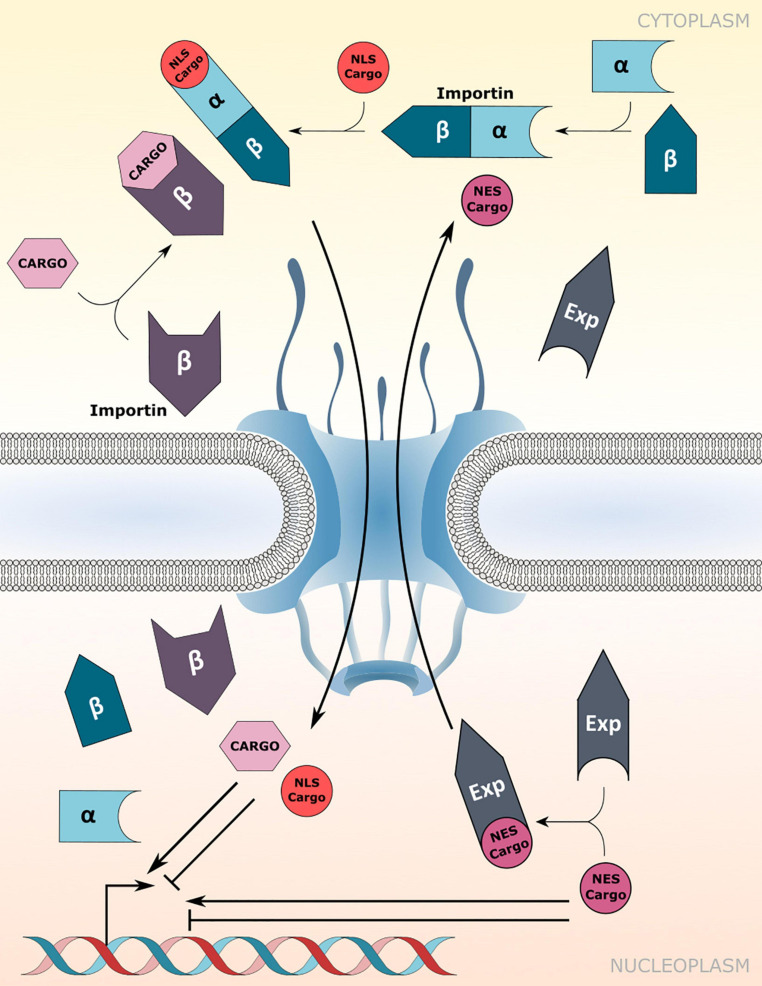
Nuclear transport receptor-mediated nucleocytoplasmic transport. Nuclear import of nuclear localization signal (NLS) containing cargo proteins *via* importin-α/β heterodimers or direct cargo-association with importin-β receptors. Nuclear export is mediated by association of exportins (Exp) with the nuclear export signal (NES) of cargo proteins. Transport directionality is indicated by curved arrows. Imported cargos or cargo destined for export may directly influence/drive transcriptional changes inside the nucleus. Experimentally verified cargos of importins and exportins are listed in Table 1.

Canonical protein transport routes into the nucleus depend on NTRs of the importin-α (IMP-α) and importin-β family (IMP-β; Christie et al., 2016). α-importins act as adaptors that recognize and bind to nuclear localization signals (NLSs) of cargo proteins. Subsequently, association of the cargo-NLS/importin-α complex with importin-β receptors enables translocation of the ternary complex through the FG-NUP permeability barrier of the NPC (Figure 1). However, importin-β receptors can recognize and import cargo proteins independently of importin-α adaptors (Figure 1; Christie et al., 2016). In addition, some karyopherin family members also bind to nuclear export signals (NESs) to facilitate the nuclear export of proteins and small RNAs, and are therefore termed exportins (Figure 1; Kutay and Güttinger, 2005).

In higher eukaryotes, there is a considerable expansion of the *importin-*α gene family (Pumroy and Cingolani, 2015). The genome of the model plant *Arabidopsis thaliana* for example encodes for nine α-importins, whereas yeast only encodes a single importin-α (Yano et al., 1992; Wirthmueller et al., 2013, 2015). This expansion might reflect adaptations toward more complex tissue- and/or stimulus-specific nuclear import mechanisms during developmental processes of higher eukaryotes and in response to environmental cues (Wirthmueller et al., 2013, 2015; Pumroy and Cingolani, 2015). Alternatively, a largely redundant repertoire of import adaptors could present a strategy to effectively buffer and protect vital signaling pathways. Indeed, the loss of a single *importin*-α gene has no obvious morphological consequences in *Arabidopsis*, while higher order mutants of *IMP-α1*/*2*/*3* display a stunted growth morphology (Chen et al., 2020; Lüdke et al., 2021). Chen et al. (2020) also reported that triple mutant plants of *IMP-α1*/*2*/*3* flower early and accordingly, the authors identified the three α-importins as adaptors that import LIKE HETEROCHROMATIN PROTEIN1 (LHP1; Table 1). LHP1 acts as a transcriptional repressor of flowering-related genes and is involved in the epigenetic regulation of developmental processes, providing an explanation for the observed flowering phenotype of the triple mutant plants (Chen et al., 2020).

**TABLE 1:** Nuclear transport receptors and their cargos.

Nuclear transport receptor	AGI code	Cargo(s)	References
IMP-α1	AT3G06720	LHP1	Chen et al., 2020
IMP-α2	AT4G16143	LHP1, PARP2	Chen et al., 2018, 2020
IMP-α3/MOS6	AT4G02150	LHP1, TN13, SNC1, BDL	Herud et al., 2016; Roth et al., 2017; Chen et al., 2020; Lüdke et al., 2021
IMP-α4	AT1G09270	IYO, PARP2	Muñoz et al., 2017; Chen et al., 2018; Contreras et al., 2019
IMP-α6	AT1G02690	BDL, PIP5K2	Herud et al., 2016; Gerth et al., 2017
KETCH1	AT5G19820	HYL1	Zhang et al., 2017
IMP-β4	AT4G27640	GIF1-3, JANUS	Liu et al., 2019; Xiong et al., 2020
SAD2	AT2G31660	MYB4	Zhao et al., 2007; Panda et al., 2020
TRN-SR/MOS14	AT5G62600	SR proteins	Xu et al., 2011
XPO1A	AT5G17020	XIW1, HDA6	Xu et al., 2019; Zhu et al., 2019
XPO1B	AT3G03110	XIW1	Xu et al., 2019
PSD/XPOT	AT1G72560	tRNAs	Hunter et al., 2003; Park et al., 2005
XPO4	AT3G04490	TPL, TPRs	Xu et al., 2021

Besides its partially redundant role with *IMP-α1* and *-α2* in plant development, a selective role of *IMP-α3* has been described in immunity (Lüdke et al., 2021). Based on the genetic requirement for the autoimmune phenotype of *suppressor of npr1−1, constitutive1* (*snc1*), a gain-of-function mutant of a nucleotide−binding leucine−rich repeat (NLR) immune receptor, *IMP-α3* was named *MODIFIER OF SNC1, 6* (*MOS6*; Palma et al., 2005). NLRs usually detect the presence or actions of immune suppressive pathogen effector molecules, but a nuclear function of SNC1 in the transcriptional regulation of defense genes and miRNAs has also been described (Zhu et al., 2010; Xu et al., 2014; Cai et al., 2018). Recent work suggests that MOS6 is the main import adapter for SNC1 (Table 1; Lüdke et al., 2021). This is consistent with the finding that the loss of *MOS6*, but not of any other *α-importin*, partially suppresses the autoimmune phenotype of *snc1* (Palma et al., 2005; Lüdke et al., 2021). In addition, the genetic requirement of *MOS6* but not of other *α-importins* for basal resistance, together with the finding that MOS6 but not its closest homolog IMP-α6 interacts with the truncated NLR TIR-NB13 (TN13), further suggests a specialization of MOS6/IMP-α3 in immunity-related cargo transport (Roth et al., 2017). In contrast, a pronounced function of IMP-α6 that partially overlaps with the function of IMP-α3 has been described for the nuclear uptake of the Aux/IAA protein BODENLOS (BDL) during primary root meristem formation (Table 1; Herud et al., 2016).

There are further examples of importin-α cargo selectivity but also of redundancy in plants (Jiang et al., 2001; Kanneganti et al., 2007; Bai et al., 2008; Gerth et al., 2017). For instance, cargo selectivity has been described for MINIYO (IYO), which interacts with IMP-α4, but not with IMP-α3 or IMP-α6 in transient assays and mass spectrometry analysis (Table 1; Muñoz et al., 2017; Contreras et al., 2019). MINIYO plays a vital role in stem cell differentiation, but no morphological phenotype has been described for *imp-α4* mutant plants, suggesting additional import routes for MINIYO (Helizon et al., 2018; Lüdke et al., 2021). On the other hand, loss of *IMP-α4* but not of other *α-importins* affects transformation of *Arabidopsis* roots by the plant pathogen *Agrobacterium tumefaciens*, although IMP-α4 and several other importin-α transport adapters are capable to interact with *Agrobacterium* effector proteins that mediate nuclear import of the T-DNA/protein complex (T-complex; Bhattacharjee et al., 2008). *IMP-α4* shows the highest expression level in roots among the *Arabidopsis importin*-α isoforms (Wirthmueller et al., 2013), indicating that IMP-α4 is the most relevant NTR for import of the T-complex and thus is exploited by *Agrobacterium* to promote plant transformation and disease progression. Importin-α mediated transport redundancy has been demonstrated for FAR-RED (FR) ELONGATED HYPOCOTYL1 (FHY1) that acts as an NLS-containing facilitator in nuclear translocation of FR light-activated phytochrome A (phyA) termed Pfr (Helizon et al., 2018). In another example, the POLY(ADP-RIBOSE) POLYMERASE2 (PARP2), that contributes to DNA-damage repair and responses to abiotic and biotic stresses, associates with multiple importin-α isoforms, but shows preferential binding to IMP-α2 and IMP-α4 (Table 1; Chen et al., 2018). The molecular basis that determines functional specialization vs. redundancy of α-importins within the divers nuclear protein import pathways remain to be experimentally dissected in plants. However, it seems plausible that in plants nuclear import kinetics and cargo recognition specificities are regulated at several layers, including different preferences for association of α- with β-importins, as well as stimulus-dependent post-translational modifications of both the NLS-cargo and the NTR(s) to either enhance or prevent importin-α/cargo interactions (Christie et al., 2016).

In contrast to defects in importin-α transport adapters, mutations in other *karyopherin* family member genes often result in pleiotropic phenotypes. This might also reflect that several α-importins redundantly depend on a specific β-importin for nuclear import of a broad cargo range. Indeed, a loss of KARYOPHERIN ENABLING THE TRANSPORT OF THE CYTOPLASMIC *HYL1* (*KETCH1*) is embryo lethal (Meinke et al., 2008). The primary miRNA processing factor HYPONASTIC LEAVES1 (HYL1), and several ribosomal proteins have been elucidated as KETCH1 imported cargos (Table 1; Zhang et al., 2017). A role in developmental processes has recently also been described for IMP-β4 which imports transcriptional regulators of *PLETHORA*, thereby regulating meristem and ovule development (Liu et al., 2019; Xiong et al., 2020). IMP-β4 also interacts with the kinesin FRAGILE FIBER1 (FRA1) to protect it from proteasomal degradation and to inhibit the motility of FRA1 by preventing its binding to microtubules. Importantly, this function of IMP-β4 appears to operate independently of its transport activity, as the binding of IMP-β4 does not lead to translocation of FRA1 into the nucleus (Ganguly et al., 2018). In *Arabidopsis*, KPNB1/IMP-β1 is involved in ABA-mediated drought stress responses, yet cargos imported by KPNB1, either directly or *via* its association with multiple α-importins, are still elusive as for most other NTRs (Luo et al., 2013; Oh et al., 2020). Pleiotropic developmental defects such as ABA-hypersensitivity and reduced trichome numbers have been reported for *super sensitive to ABA and drought2* (*sad2*) mutants (Verslues et al., 2006; Gao et al., 2008; Yoshida et al., 2009). SAD2 also mediates nuclear import of MYB4, a transcriptional repressor of the phenylpropanoid metabolism that regulates lignin biosynthesis (Zhao et al., 2007; Panda et al., 2020). In addition, SAD2 acts as a negative regulator of miRNA pathways and functions in Ca^2+^- and reactive oxygen species (ROS)-mediated cell death responses, arguing for its involvement in several signaling pathways and physiological responses (Wang et al., 2011; Zheng et al., 2020). The discovery of an additional *MOS* gene, *MOS14*, that encodes for the importin-β superfamily protein TRANSPORTIN (TRN)-SR, further outlines the importance of the nucleocytoplasmic trafficking machinery for plant immune responses. The loss of *MOS14* influences the splicing patterns of *NLR* transcripts, including *SNC1*, due to reduced nuclear accumulation of serine-arginine rich (SR) proteins, which are required for splice site recognition and spliceosome assembly (Xu et al., 2011).

The involvement in several signaling pathways has also been described for EXPORTIN1 (XPO1). *Xpo1a* mutant plants show an impaired heat-stress response, while *xpo1a xpo1b* double mutant plants are lethal due to defects in gametogenesis (Blanvillain et al., 2008; Wu et al., 2010). *XPO1A* and *XPO1B* are further required in the ABA response pathway and were shown to be export factors for a positive regulator of the ABA-response, named XPO1-Interacting WD40 protein1 (XIW1; Table 1; Xu et al., 2019). In addition, HISTONE DEACETYLASE6 (HDA6), a transcriptional gene silencing factor, interacts with XPO1A and accumulates in nuclei of *xpo1a* mutant plants, further demonstrating the pleiotropic role of XPO1A in nuclear export (Table 1; Zhu et al., 2019). Mutants of the *Arabidopsis* exportins *PAUSED* (*PSD*)/*EXPORTIN-T* and *HASTY* (*HST*)/*EXPORTIN5* display a delay or acceleration in a range of developmental processes, respectively (Bollman et al., 2003; Hunter et al., 2003; Li and Chen, 2003). While PSD exports tRNAs, *hst* mutants show reduced miRNA levels without affecting the subcellular distribution of miRNAs (Park et al., 2005; Bologna et al., 2018; Cambiagno et al., 2020). Since HST interacts with proteins of the mediator (MED) complex that is part of the transcriptional machinery, HST might have a function as an export-independent scaffold for transcription and processing of primary miRNA transcripts (Cambiagno et al., 2020).

Overall, the number of elucidated NTR cargos and interaction partners remains limited. This might be explained by the transient nature of the interaction between NTRs and their cargo clients. Xu et al. (2021) recently demonstrated that the use of proximity-based labeling approaches provides exciting new possibilities for the identification of cargo-NTR associations in plants. Using TurboID-tagged variants of several exportins, the authors provide evidence for a selective interaction of EXPORTIN4 (XPO4) with members of the TOPLESS (TPL) and TPL-related (TPR) protein family (Table 1). Consistent with the observation that the loss of *XPO4* function enhances autoimmunity of the nucleoporin mutant *cpr5* (*constitutive expresser of PR genes5*), the authors show that the export activity of XPO4 counteracts nuclear accumulation of TPL and TPRs, which are involved in transcriptional co-repression of negative regulators of immunity (Zhu et al., 2010; Xu et al., 2021). The modulation of distinct signaling pathways observed in *cpr5* and other nucleoporin mutants outlines the importance and active contribution of NPC components in the regulation of nuclear translocation processes.

### Nucleoporin Functions in Protein Transport and Homeostasis

**FIGURE 2:**
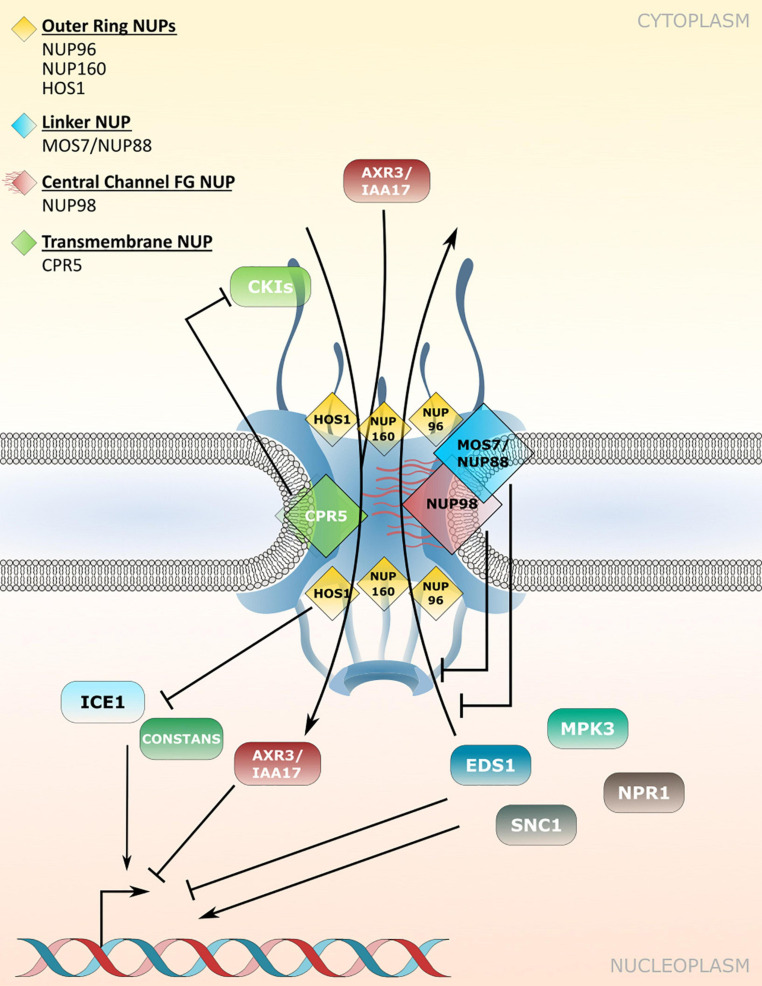
Nucleoporin and NPC-associated protein functions in nucleocytoplasmic protein transport. Several nucleoporins regulate protein transport through the NPC. CPR5 controls influx of cyclin-dependent kinase inhibitors (CKIs) during effector-triggered immunity (ETI), while MOS7/NUP88 associates with the FG-repeat containing NUP98 to attenuate nuclear efflux of several immunity related proteins. The NUP107-160 complex members NUP96 and NUP160 associate with HOS1 and regulate the nuclear protein abundance of temperature and flowering associated factors. Several NUP regulated cargos influence transcriptional responses in the nucleus. Table 2 lists nucleoporin functions in protein transport and the respective *nucleoporin* mutant phenotypes.

CRP5 is a plant-specific transmembrane nucleoporin which forms homomeric complexes and associates with the core scaffold of the NPC in steady-state tissues (Gu et al., 2016). Upon activation of effector-triggered immunity (ETI) by intracellular NLR immune receptors, CPR5 undergoes a conformational switch that disrupts CPR5 oligomer formation and hence the selective barrier of the NPC. This is considered to allow massive nuclear import of ETI-related signaling cargos, including cyclin-dependent kinase inhibitors (CKIs) that dissociate from the NPC upon CPR5 monomerization to enable the expression of immune response genes (Figure 2 and Table 2; Wang et al., 2014; Gu et al., 2016). Consistent with a negative regulatory role of CPR5 in ETI by preventing uncontrolled nuclear influx of stress signaling cargos, *cpr5* mutant plants show an autoimmune phenotype, whereas overexpression of CRP5 compromises resistance and ETI-associated programmed cell death (PCD; Wang et al., 2014; Gu et al., 2016).

**TABLE 2:** Nucleoporin and NPC-associated protein functions in nucleocytoplasmic protein transport.

Nucleoporin	AGI code	Cellular functions (mutant phenotypes)	References
CPR5	AT5G64930	ETI-triggered CPR5-monomerization enables nuclear influx of CKIs; (*cpr5*: autoimmunity)	Wang et al., 2014; Gu et al., 2016
NUP88/MOS7	AT5G05680	Attenuates nuclear export rates of EDS1, SNC1, NPR1, MPK3; (*mos7-1*: increased susceptibility to diverse pathogens; het. *mos7-5*: ovule and pollen abortion; lethality of *mos7* null alleles)	Cheng et al., 2009; Park et al., 2014; Genenncher et al., 2016
NUP98A/DRA2	AT1G10390	Interacts with MOS7 to regulate plant immunity; involved in SAS regulation; positive regulator of starch degradation; (*nup98a*: increased susceptibility to *B. cinerea*, *nup98a1 nup98b1*: early flowering, early senescence)	Gallemí et al., 2016; Genenncher et al., 2016; Jiang et al., 2020; Xiao et al., 2020
NUP98B	AT1G59660	Interacts with MOS7; positive regulator of starch degradation; (*nup98a1 nup98b1*: early flowering, early senescence)	Genenncher et al., 2016; Jiang et al., 2020; Xiao et al., 2020
HOS1	AT2G39810	Mediates degradation of CONSTANS and ICE1; (*hos1*: early flowering, enhanced cold stress tolerance, nuclear accumulation of PIF4 at elevated temperature)	Dong et al., 2006a; Cheng et al., 2020; Li et al., 2020; Zhang A. et al., 2020
NUP160/SAR1	AT1G33410	Stabilization of HOS1 at NPC; (*nup160*: early flowering, reduced cold stress tolerance, reduced nuclear accumulation of AXR3/IAA17)	Dong et al., 2006b; Parry et al., 2006; Wiermer et al., 2012; Li et al., 2020
NUP96/MOS3/SAR3	AT1G80680	Stabilization of HOS1 at NPC; (*nup96*: early flowering, reduced nuclear accumulation of AXR3/IAA17, nuclear accumulation of PIF4 at elevated temperature)	Parry et al., 2006; Cheng et al., 2020; Zhang A. et al., 2020;

The modulation of plant immune responses at the level of protein translocation across the NPC has also been revealed for *Arabidopsis* NUP88/MOS7 and significantly, the autoimmune phenotype of *cpr5* is suppressed by the partial loss-of-function mutation *mos7-1*, causing a four amino acid deletion (Cheng et al., 2009; Wiermer et al., 2010; Genenncher et al., 2016; Gu et al., 2016). NUP88/MOS7 attenuates nuclear export rates of important nucleocytoplasmic defense proteins, such as the regulator of pattern-triggered basal defense and TIR-type NLR-mediated immunity ENHANCED DISEASE SUSCEPTIBILITY1 (EDS1), and NONEXPRESSOR OF PATHOGENESIS-RELATED GENES1 (NPR1) that functions as a receptor for the plant defense hormone salicylic acid (SA; Figure 2 and Table 2; Mou et al., 2003; Feys et al., 2005; Cheng et al., 2009; García et al., 2010; Genenncher et al., 2016). The genetic dependency of *cpr5* autoimmunity on *MOS7* implies that a MOS7-mediated nuclear retention of common stress signaling cargos is required for (auto)immunity and PCD upon CPR5 gated nuclear cargo influx. Indeed, Gu et al. (2016) showed that the overexpression of CPR5 constrains the nuclear translocation of NPR1 as well as of other stress- and plant hormone-related nuclear signaling cargos. These examples suggest that nuclear protein influx and efflux are regulated by distinct NPC constituents to mount a robust immune response.

Consistent with the phenotype of *nup88* mutations in other organisms, null alleles of *Arabidopsis mos7* are lethal, suggesting that in addition to its function in regulating a diverse set of plant immune responses, wild-type MOS7/NUP88 is also essential for regular plant growth and development (Cheng et al., 2009; Wiermer et al., 2010). Accordingly, work by Park et al. (2014) implicates MOS7/NUP88 in mitosis during female and male gametophyte formation and seed development. Using forward genetics, the authors identified *mos7-5*, which results in ovule and pollen abortion in heterozygous *mos7-5*/*MOS7* plants. Whereas MOS7 localizes to the NE during interphase, it associates with mitotic microtubules during cell division, suggesting additional transport-independent functions of MOS7 in microtubule organization and dynamics (Park et al., 2014). NUP88/MOS7 interacts with the FG-NUPs NUP98A and NUP98B which appears to be essential to regulate the permeability of the NPC for certain immune regulatory cargo proteins (Figure 2; Cheng et al., 2009; Genenncher et al., 2016). Indeed, *nup98a* mutant plants are more susceptible to *Botrytis cinerea* infection (Genenncher et al., 2016). Consistent with immune-regulatory roles of NUP98 in *Arabidopsis*, its putative homolog in rice, APIP12, is required for resistance to the rice blast fungus *Magnaporthe oryzae* and is targeted by the *M. oryzae* effector AvrPiz-t (Tang et al., 2017).

In *Arabidopsis*, additional roles of NUP98 in plant environmental responses and development have been revealed. For example, *NUP98A* was identified in a genetic screen for regulatory components of the shade avoidance syndrome (SAS) and has been termed DRACULA2 (DRA2; Table 2; Gallemí et al., 2016). NUP98A/DRA2 appears to be a dynamic nucleoporin, as it localizes to the nuclear rim as well as to the cytoplasm and the nucleoplasm when transiently overexpressed (Gallemí et al., 2016). It is currently unknown whether the SAS phenotype of *dra2* mutant plants is caused by light-dependent alterations in the nucleocytoplasmic translocation of light signal transducers such as phytochrome photoreceptors, and/or based on its defect in nuclear mRNA export (Gallemí et al., 2016). However, its cellular distribution may provide NUP98A/DRA2 with additional transport-independent functions, such as the regulation of shade-induced gene expression *via* associations with chromatin or chromatin-bound transcription complexes, as was proposed by Gallemí et al. (2016). Consistent with this idea, metazoan NUP98 is mobile and has multiple reported functions, including the regulation of gene expression *via* direct associations with chromatin (Griffis et al., 2002; Kalverda et al., 2010). *Arabidopsis* NUP98A and NUP98B also function redundantly as positive regulators of starch degradation, thus delaying plant senescence (Xiao et al., 2020), and act as negative regulators of flowering in a *CONSTANS* (*CO*) transcriptional regulator-independent manner (Jiang et al., 2020).

The early flowering phenotype of *nup98a nup98b* double mutants is common to several other mutants of nucleoporin encoding genes, including *NUP160* and *NUP96*, two members of the NUP107-160 nuclear pore sub-complex (Table 2; Dong et al., 2006b; Parry et al., 2006; Jacob et al., 2007; Tamura et al., 2010). Whereas this phenotype might be related, at least in part, to defects in nuclear mRNA export (see chapter below), additional defects in protein stability and nuclear protein transport may contribute to the mutant phenotypes. In the case of NUP160 and NUP96, it was reported recently that both nucleoporins associate with and stabilize the E3-ubiquitin ligase HIGH EXPRESSION OF OSMOTICALLY RESPONSIVE GENES1 (HOS1) at the NPC (Figure 2 and Table 2), which negatively regulates flowering transition *via* ubiquitination and subsequent proteasomal degradation of CO (Cheng et al., 2020; Li et al., 2020). Loss of either *HOS1*, *NUP96* or *NUP160* results in nuclear accumulation of CO and subsequent CO-mediated transcriptional activation of *FLOWERING LOCUS T* (*FT*). FT induces the expression of genes that contribute to the formation of floral primordia, providing an explanation for the early flowering phenotypes of *hos1*, *nup96* and *nup160* plants (Cheng et al., 2020; Li et al., 2020).

HOS1 also interacts with the transcription factor ICE1 and mediates its degradation in order to attenuate cold responses in *Arabidopsis* (Figure 2 and Table 2; Dong et al., 2006a). While both, *hos1* and *nup160* plants, show an early flowering phenotype, the two mutants show opposite – i.e., enhanced (*hos1*) and reduced (*nup160*) – tolerance to cold stress (Dong et al., 2006a,b). Considering that NUP96 and NUP160 promote the stabilization and association of HOS1 at the NPC during flowering regulation (Cheng et al., 2020; Li et al., 2020), a scenario of mutual NUP160-HOS1 stabilization at the NPC appears unlikely in cold stress signaling, and might be attributed to the dynamic nuclear accumulation of HOS1 in response to low temperature (Lee et al., 2001). However, the localization of HOS1 has not been investigated in *nup160* or *nup96* plants grown under ambient or chilling/freezing conditions. While the nuclear localization of ICE1 is not obviously affected in *nup160* plants (Dong et al., 2006b), *nup160* (also termed *sar1* for *suppressor of auxin resistance1*) and *nup96* (also termed *sar3*) show reduced nuclear accumulation of the transcriptional repressor AXR3/IAA17 (Figure 2 and Table 2), which may cause the altered auxin-dependent responses of *nup160* and *nup96* (Parry et al., 2006). This suggests a different extent to which NUP160 and NUP96 modulate the nuclear abundance of protein regulators to coordinate various signaling pathways in response to environmental and developmental cues. This includes adaptations to elevated temperatures mediated by the transcription factor PHYTOCHROME INTERACTING FACTOR4 (PIF4; Zhang A. et al., 2020), but could also involve additional functions of these NUP107-160 complex members in nuclear mRNA export.

### Nucleoporin Functions in mRNA Transport and Metabolism

**FIGURE 3:**
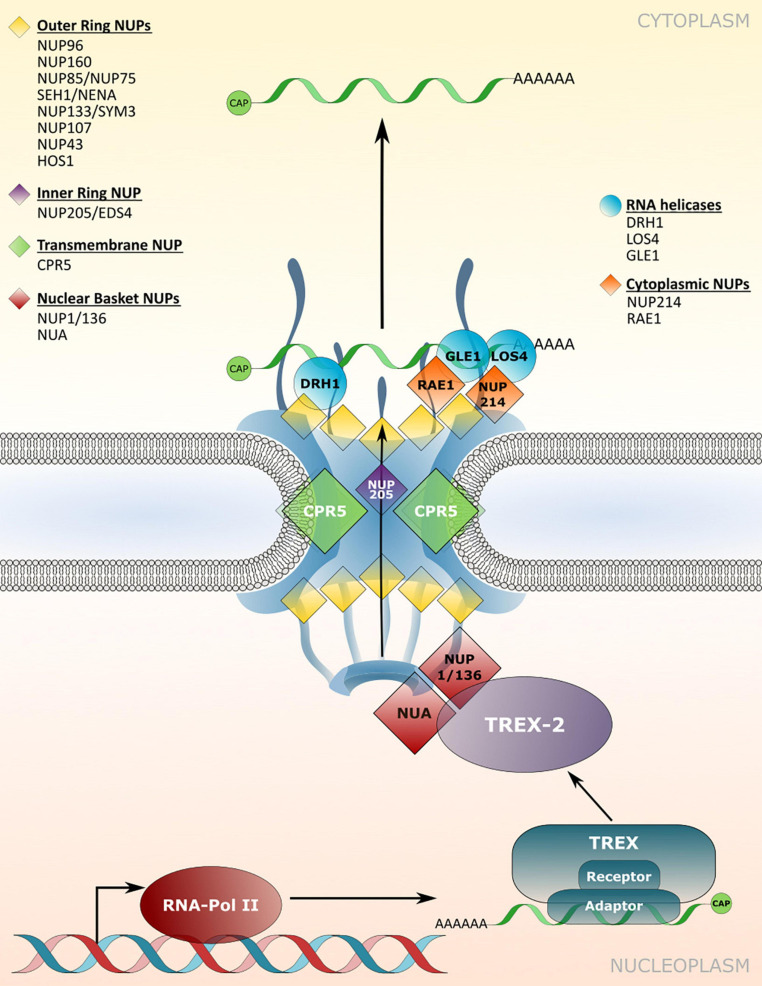
Nucleoporin and NPC-associated protein functions in mRNA transport. Several nucleoporins are required for the efficient translocation of mRNAs/mRNPs through the NPC, which involves members of TREX/TREX-2 complex and RNA binding adaptor proteins. mRNP remodeling by NPC-associated RNA helicases at the cytoplasmic site ensures unidirectionality of transport. Nucleoporins involved in nuclear mRNA export and the respective mutant phenotypes are listed in Table 3.

The efficient nuclear export of mature mRNAs *via* mRNPs to the cytoplasmic translation machinery is a crucial step in cellular responses initiated upon the integration of endogenous and exogenous signals inside the nucleus, which direct transcriptional changes. A central part of mRNP export is the transcription-export (TREX) complex, which associates with transcripts and aids in the recruitment of mRNA export adaptors and receptors (Figure 3; Ehrnsberger et al., 2019; Ashkenazy-Titelman et al., 2020). Transport through the permeability barrier of the NPC involves the interaction of mRNPs with the TREX-2 complex that is tethered to the nuclear basket of the NPC (Figure 3). While mRNA adaptors remain inside the nucleus, mRNA export receptors are released upon translocation through the NPC and mRNP remodeling *via* RNA helicases at the cytosolic side of the NPC (Ehrnsberger et al., 2019; Ashkenazy-Titelman et al., 2020). Components required for mRNP translocation across the NPC are well described in yeast and mammalian systems, but knowledge on mRNP translocation in plants is limited. Although several mRNA export adaptors and TREX complex homologues have been identified in *Arabidopsis*, prime candidates for plant homologs of the mRNA export receptor are still elusive, suggesting plant-specific export components/mechanisms for this critical step (Ehrnsberger et al., 2019). A loss of mRNA export adaptors, TREX or TREX-2 complex members in *Arabidopsis* can lead to nuclear mRNA accumulation and affects several developmental and stress response pathways, including plant immunity (Germain et al., 2010; Pan et al., 2012a,b; Sørensen et al., 2017; Uddin et al., 2017; Ehrnsberger et al., 2019). Nuclear mRNA accumulation has also been observed upon mutation of several *nucleoporin* genes, although the direct molecular functions of most nucleoporins in mRNA export have not been revealed. In addition, the identified nucleoporins can have multiple functions in the translocation of mRNAs and proteins, and may also possess additional transport-independent functions (Ehrnsberger et al., 2019; see chapters on nucleoporins in protein transport, and on transport-independent nucleoporin functions), which could also account for pleiotropic defects observed for some of the *nucleoporin* mutants.

The nuclear basket localized *Arabidopsis* nucleoporins NUP136/NUP1 and NUA interact with members of the TREX-2 complex, implicating these components as the first contact site of mRNPs with the plant NPC for translocation (Figure 3; Lu et al., 2010; Yang et al., 2017; Zhang B. et al., 2020). Consistent with this function, a loss of *NUP1* and *NUA* leads to nuclear mRNA accumulation and mutant plants display defects in gametogenesis as well as an early flowering phenotype, while *nua* mutant plants also show defects in miRNA export (Table 3; Xu et al., 2007; Lu et al., 2010; Tamura et al., 2010; Bao et al., 2019; Zhang B. et al., 2020). Early flowering phenotypes combined with nuclear mRNA accumulation can also be observed in mutant plants with defects in different NUP107–160 complex members such as *NUP160* and *NUP96*, whereas mutants of other complex members like *NUP85* or *SEH1* do not flower early but also accumulate mRNA inside nuclei (Figure 3 and Table 3; Dong et al., 2006b; Parry et al., 2006; Wiermer et al., 2012; MacGregor et al., 2013; Parry, 2014; Du et al., 2016). This suggests that defects in nuclear mRNA export *per se* do not cause the mutant plants to flower early. Therefore, certain nucleoporins may be involved in nuclear export of distinct sub-pools of mRNAs, or may possess additional functions that are not related to mRNA transport, thus causing particular mutant phenotypes. Since nucleoporins such as the NUP107–160 complex members associate in larger sub-complexes within the NPC (Tamura et al., 2010; Cheng et al., 2020), it can be speculated whether an overall compromised structural integrity of the NPC is the major reason for bulk mRNA accumulation inside the nucleus of the respective single mutant plants. In such a scenario, the loss of some nucleoporins, like NUP160 or NUP96, may have a stronger impact on the functionality and structural integrity of the NPC compared to mutations in other complex members. This might either be due to multiple interactions with other members of the sub-complex, or because of (partially) redundant functions among the different sub-complex members. In addition, NUP96 protein levels are reduced in *hos1* mutant plants (Cheng et al., 2020), further suggesting that the structural integrity of the NPC is also a prerequisite for the stabilization of other NPC constituents that play a role in mRNA export.

**TABLE 3:** Nucleoporin and NPC-associated protein functions in mRNA transport.

Nucleoporin	AGI code	Cellular functions (mutant phenotypes)	References
NUP1/136	AT3G10650	Interaction with THP1/TREX-2 members; (*nup1/136:* nuclear mRNA accumulation, early flowering, defects in gametogenesis)	Lu et al., 2010; Tamura et al., 2010; Bao et al., 2019; Zhang B. et al., 2020
NUA	AT1G79280	Interaction with TREX-2 member SAC3B; (*nua*: nuclear mRNA accumulation, early flowering, defects in miRNA export)	Jacob et al., 2007; Xu et al., 2007; Yang et al., 2017
NUP160/SAR1	AT1G33410	Essential for autoimmune phenotypes of *snc1* and *bak1 bkk1*; (*nup160*: nuclear mRNA accumulation, early flowering, increased susceptibility)	Dong et al., 2006b; Parry et al., 2006; Wiermer et al., 2012; Du et al., 2016
NUP96/MOS3/SAR3	AT1G80680	Essential for autoimmune phenotypes of *snc1* and *bak1 bkk1*; (*nup96*: nuclear mRNA accumulation, early flowering, increased susceptibility)	Parry et al., 2006; Wiermer et al., 2012; Du et al., 2016; Cheng et al., 2020
SEH1	AT1G64350	Essential for autoimmune phenotypes of *snc1* and *bak1 bkk1*; (*seh1*: nuclear mRNA accumulation, increased susceptibility)	Wiermer et al., 2012; Du et al., 2016
NUP85	AT4G32910	Essential for autoimmune phenotype of *bak1 bkk1*; (*nup85*: nuclear mRNA accumulation)	Du et al., 2016
NUP214	AT1G55540	Interaction with DEAD-BOX RNA helicase LOS4; (*nup214*: embryo lethality)	Braud et al., 2012
RAE1	AT1G80670	Interaction with DEAD-BOX RNA helicase GLE1	Tamura et al., 2010
HOS1	AT2G39810	Interaction with RAE1 and NUP85; (*hos1*: nuclear mRNA accumulation)	Tamura et al., 2010; MacGregor et al., 2013; Zhu et al., 2017
CPR5	AT5G64930	(*cpr5*: nuclear accumulation of mRNAs encoding ethylene signaling factors)	Chen et al., 2019
NUP205/EDS4	AT5G51200	(*eds4*: nuclear accumulation of mRNAs encoding circadian clock and immunity related factors)	de Leone et al., 2020

Homologs of yeast/mammalian DEAD-BOX RNA helicases implicated in mRNP remodeling at the cytoplasmic side of the NPC have also been identified in *Arabidopsis*, which includes LOS4 and GLE1 (Gong et al., 2002; Tamura et al., 2010). Accordingly, both proteins are associated with nucleoporins located on the cytoplasmic side of the NPC (Figure 3). While LOS4 interacts with NUP214, GLE1 has been found in complex with the NPC associated RNA EXPORT FACTOR1 (RAE1; Tamura et al., 2010; Braud et al., 2012). Null mutants of *NUP214* and *GLE1* are embryo lethal (Braud et al., 2012), while a point mutation in *LOS4* leads to mRNA accumulation inside the nucleus and enhanced chilling and freezing stress tolerance, but decreased heat stress tolerance (Gong et al., 2002; Lee et al., 2015). GLE1 also interacts with LOS4 and thereby positively regulates cold stress responses in a phytic acid (insP6) dependent manner, outlining a central role of these nucleoporins in plant responses to temperature changes (Lee et al., 2015).

While most studies report on bulk mRNA accumulation inside nuclei upon mutation of individual nucleoporins, transcript specific export defects have also been revealed. Chen et al. (2019) recently reported that a loss of *CPR5* leads to nuclear accumulation of several transcripts encoding for components of the ethylene signaling pathway, whereas *nup96* and *nup160* seedling nuclei appear to accumulate a broader or different set of transcripts. In addition, de Leone et al. (2020) disclosed that mutations *in EDS4/NUP205*, which lead to protein truncation, result in mRNA accumulation, specifically affecting transcripts of circadian clock and immunity related genes (Table 3). Given that several other nucleoporins have been shown to be required for distinct immune responses, it is tempting to speculate that a subset of mRNAs whose nuclear export is regulated by defense-related nucleoporins encode for proteins implicated in the regulation of particular defense pathways. Although such pathway specificity may also be attributed to additional nucleoporin functions in protein transport or in transport-independent gene-regulatory functions (see chapter below), analyses in other model organisms revealed a preferential association of certain RBPs and export adapters with distinct classes of functionally related mRNAs, indicating that mRNA export pathways are not identical (Hieronymus and Silver, 2003; Kim Guisbert et al., 2005; Köhler and Hurt, 2007). Therefore, distinct nucleoporins may coordinate the nuclear translocation of specific sets of mRNAs by modulating the interaction of mRNPs with the NPC (Moore, 2005; Chakraborty et al., 2008), potentially explaining differences in the sensitivity of certain signaling pathways to the disruption of particular nucleoporins.

Recently, a function of the NUP107–160 complex was disclosed in autoimmunity and spontaneous cell death activated by the simultaneous loss of the receptor-like kinases (RLKs) BRI1-ASSOCIATED KINASE1 (BAK1) and its closest homolog BAK1-LIKE1 (BKK1; Table 3). Du et al. (2016) show that the *bak1 bkk1* double mutant phenotypes genetically depend on *NUP160*, *NUP96*, *NUP85*, and *SEH1*, as well as the NUP107–160 complex interacting *DEAD BOX RNA HELICASE1* (*DRH1*) that is required for nuclear mRNA export (Du et al., 2016). In *Nicotiana benthamiana*, silencing of the *NUP85* homolog *NUP75* affects late defense responses to the oomycete pathogen *Phytophthora infestans* (Ohtsu et al., 2014). Intriguingly, NUP85 and the NUP107-160 complex members NUP133/SYM3 and SEH1/NENA are also essential for symbiotic interactions of the model legume *Lotus japonicus* (Kanamori et al., 2006; Saito et al., 2007; Groth et al., 2010), substantiating an important role of this nuclear pore sub-complex in plant responses to both symbiotic and pathogenic microorganisms. Whether these functions are regulated by specific signaling events and based on the transport of mRNAs or proteins or on transport-independent functions, or a combination of these processes is a future challenge to address.

### Transport-Independent Functions of NPCs and NE-Associated Proteins in Gene Expression and Chromatin Organization

Transcriptional adaption is a key process in initiating cellular responses to developmental as well as biotic and abiotic signals. Apart from functions in protein and RNA transport processes described above, NPCs together with NE-associated proteins are also involved in the spatial chromatin organization and mediate gene positioning at the nuclear periphery to regulate gene expression and promote mRNA export. The idea of NPC-mediated gene regulation was already proposed more than 35 years ago in the “gene gating” hypothesis (Blobel, 1985). Indeed, the sub-nuclear localization of chromatin is correlated to the degree of gene expression, and several examples in yeast and vertebrates provide evidence for direct interactions of nucleoporins with actively transcribed chromatin (Van Bortle and Corces, 2012; Ibarra and Hetzer, 2015).

**FIGURE 4:**
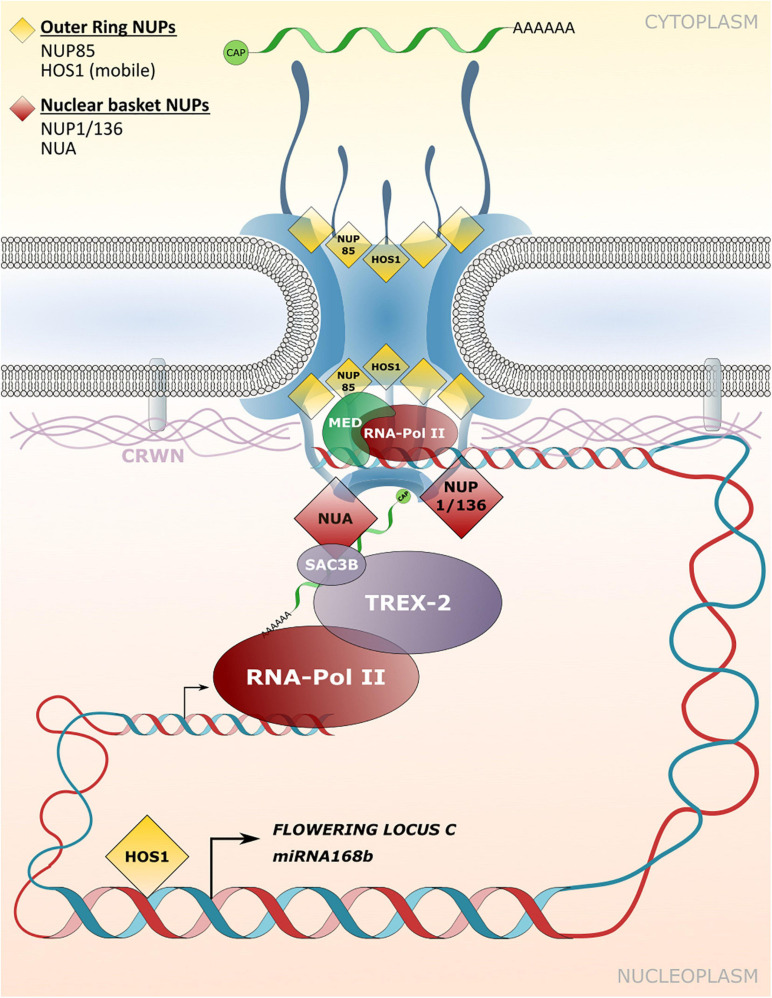
Transport independent functions of NPCs and NE-associated protein functions in gene expression and chromatin organization. Nucleoporins and nuclear matrix constituent proteins such as CROWDED NUCLEI (CRWN) in *Arabidopsis* that are implicated in tethering chromatin regions to the NPC/INM or in associating with transcriptional regulators are shown. These interactions influence transcriptional responses independently of – or in addition to – NPC functions in macromolecular transport between the cytoplasm and the nucleoplasm.

In plants, limited examples of direct associations between nucleoporins and chromatin have been described. The NUP107-160 complex associated HOS1 was shown to interact with the chromosomal *FLOWERING LOCUS C* (*FLC*) that acts as a flowering repressor (Figure 4; Jung et al., 2013). Accordingly, mutation of *HOS1* results in reduced *FLC* gene expression in *Arabidopsis* plants and the early induction of flowering (Lee et al., 2001; Jung et al., 2013). The *FLC* chromatin binding efficiency to HOS1 is strongly elevated under cold stress conditions and requires FVE, which usually acts as a negative regulator of *FLC* gene expression (Kim et al., 2004; Jung et al., 2013). The HOS1-FVE complex formed under cold stress inhibits the chromatin binding of HISTONE DEACETYLASE6 (HDA6) that is required for silencing of *FLC*. The chromatin association of HOS1 therefore leads to suppression of flowering initiation under cold stress (Jung et al., 2013). In another example, HOS1 was shown to positively regulate the expression of *miRNA168b* by associating with chromatin of the *miRNA168b* promoter region. *miRNA168b* targets *ARGONAUTE1* transcripts, and thus HOS1 may be involved in influencing the activity of the RNA-induced silencing machinery (Wang et al., 2015). HOS1 also associates with members of the NUP107-160 complex, such as NUP85 and NUP160 (Figure 4; Zhu et al., 2017; Cheng et al., 2020), and recently all three nucleoporins were identified as positive regulators of abscisic acid (ABA) and salt stress responses in *Arabidopsis* (Zhu et al., 2017). Consequently, *nup85*, *hos1* and *nup160* mutant plants are hypersensitive to ABA and salt stress and display strongly impaired expression of the respective stress-related genes (Zhu et al., 2017). It is currently not known whether the nucleoporins of the NUP107–160 complex directly interact with and tether chromatin regions of these stress-related genes to the NPC. Strikingly, subunits of the MED core transcriptional machinery complex associate with NUP85 and directly link RNA polymerase II-mediated transcriptional regulation to this NPC component (Figure 4; Zhu et al., 2017). Further evidence for a positive regulatory function of the NUP107–160 complex in gene expression has been provided by Smith et al. (2015). Artificial tethering of a reporter gene construct to the nuclear periphery *via* interaction with the NUP107–160 component SEH1 resulted in induced expression of the reporter construct (Smith et al., 2015). In addition, and consistent with immunity defects of *nup160* plants, Wiermer et al. (2012) showed that *Arabidopsis NUP160* is required for full gene expression of the defense regulator *EDS1*, but a direct involvement of NUP160 in *EDS1* transcriptional regulation at the NPC is unknown.

A potential function in tethering of chromatin to the NPC has also been described for nucleoporins that localize to the nuclear basket. NUA directly associates with SAC3B, a member of the TREX-2 complex, and both components are required for mRNA export (Figure 4; Xu et al., 2007; Yang et al., 2017). However, a loss of *NUA* and *SAC3B* also leads to reduced transcript abundance of a genome-integrated reporter gene construct (Yang et al., 2017). The authors characterize SAC3B as an anti-silencing factor that prevents heterochromatin formation as an epigenetic silencing mechanism, and potentially tethers chromatin regions to the NPC *via* its interaction with NUA for enhanced transcriptional activity (Yang et al., 2017).

Based on the reduced expression of reproduction-related genes in *nup1/136* mutant plants, Bao et al. (2019) speculate that NUP1/136 directly regulates gene expression *via* the recruitment of chromatin regions to the NPC. A strongly reduced expression of SA-responsive genes such as the defense marker *PATHOGENESIS-RELATED GENE1* (*PR1*) has also been described for *nup1/136* as well as *nup82* mutant plants, further supporting a role in transcriptional regulation of these nucleoporins (Tamura et al., 2017). Using restriction enzyme-mediated chromatin immunoprecipitation (RE-ChIP) assays with NUP1/136-GFP as bait, Bi et al. (2017) demonstrated that NUP1 is primarily associated with transcriptionally repressed chromatin region. However, some highly expressed genes were also enriched in NUP1/136-GFP precipitated chromatin regions, consistent with a subset of these genes showing reduced expression in *nup1/136* mutant plants (Bi et al., 2017; Tamura et al., 2017). This suggests a different degree to which gene expression can be modulated by NUP1/136 and may thus involve additional factors that associate with a particular chromosomal locus.

Detailed studies in animal systems have demonstrated that repressed chromatin regions found at the nuclear periphery often associate with lamins and are therefore termed lamin-associated domains (LADs; Pickersgill et al., 2006; Guelen et al., 2008; Ikegami et al., 2010; Peric-Hupkes et al., 2010; van Steensel and Belmont, 2017). Lamins are part of the nucleoskeleton at the inner side of the NE, interact with NE localized membrane proteins and are functionally required in determining nuclear shape and nuclear positioning (Groves et al., 2020). Although plants do not encode for canonical lamins, plant specific structural and functional equivalents can be found and have been termed nuclear matrix constituent proteins (NMCPs; Dittmer et al., 2007; Sakamoto and Takagi, 2013; Wang et al., 2013; Ciska and Moreno Díaz de la Espina, 2014; Kimura et al., 2014; Meier et al., 2017; Sakamoto et al., 2020). In *Arabidopsis*, NMCPs considered to fulfill lamin-like functions are named CROWDED NUCLEI (CRWN) 1–4 (Dittmer et al., 2007; Wang et al., 2013). Indeed, CRWN1 directly interacts with INM associated proteins and, like CRWN2–4, can be found at the nuclear periphery where these proteins form a meshwork structure (Figure 4), while CRWN2 and CRWN3 are also present in the nuclear interior (Dittmer et al., 2007; Sakamoto and Takagi, 2013; Graumann, 2014; Sakamoto et al., 2020). As for their animal equivalents, CRWN proteins play a role in determining the nuclear shape since *crwn* mutant plants display abnormally shaped or small nuclei (Wang et al., 2013). A role in tethering heterochromatin regions to the NE – which were previously also identified by ChIP assays with NUP1/136-GFP – has been demonstrated for CRWN1 and CRWN4, providing a potential link between the NPC and plant lamin-like proteins in positioning chromatin at the nuclear periphery (Hu et al., 2019). Significantly, CRWN1 and CRWN4 can also be found in complex with PROLINE-TRYPTOPHANE-TRYPTOPHANE-PROLINE (PWWP) INTERACTOR OF POLYCOMBS1 (PWO1), a component of the repressive Polycomb-Group (PcG) complex that associates with repressed chromatin regions, further outlining a role of these CRWN proteins in gene repression in plants (Mikulski et al., 2019).

It has been reported that CRWN1 directly interacts with the transcription factor NAC WITH TRANSMEMBRANE MOTIF1-LIKE9 (NTL9) and the transcriptional repressor SUPPRESSOR OF NPR1-1, INDUCIBLE1 (SNI1), thereby repressing *PR1* gene expression (Guo et al., 2017). Consequently, *crwn1* and *crwn1 crwn2* double mutant plants show elevated defense gene expression, leading to enhanced resistance to *Pseudomonas* bacteria (Guo et al., 2017). Similarly, Choi et al. (2019) reported an enhanced *PR1* gene expression in *crwn1 crwn2* as well as in *crwn1 crwn4* double mutant plants. Both double mutant lines display enhanced resistance to *Pseudomonas* bacteria, albeit no enhanced resistance was observed for either of the single mutant lines. Strikingly, *crwn1 crwn2* and *crwn1 crwn4* double mutants show elevated levels of SA due to the enhanced expression of SA biosynthesis genes and master transcription factors regulating genes of the SA-defense pathway (Choi et al., 2019). In *crwn1 crwn2*, this correlated with reduced histone modifications in chromatin regions encoding for a subset of these defense genes, further arguing for a direct repression of chromatin regions *via* CRWN-mediated tethering to the nuclear periphery (Choi and Richards, 2020).

It is noteworthy that Choi et al. (2019) consistently observed intermediate defense-associated phenotypes of the *crwn1 crwn4* double mutant when compared to wild-type plants and the *crwn1 crwn2* mutant, outlining differential and – on global gene expression patterns – partially antagonistic functions for the CRWN proteins on transcription. In another recent study, *crwn1* mutants show elevated levels of jasmonic acid (JA), a plant hormone required for defense against necrotrophic pathogens that kill host tissues (Jarad et al., 2020). This molecular phenotype of *crwn1* mutant plants correlates with enhanced resistance toward the necrotrophic fungus *B. cinerea* (Jarad et al., 2020). In contrast, *crwn1* plants are more susceptible to non-virulent *Pseudomonas* bacteria and show defects in early defense responses (Jarad et al., 2020). These results outline the important role of plant lamin-like proteins in the transcriptional regulation of multiple defense pathways.

Apart from the effect on immune responses, mutations in *CRWN* genes also affect developmental processes such as seed germination, since *crwn1 crwn3* double mutant plants show reduced germination rates and are hypersensitive to ABA (Zhao et al., 2016). The authors demonstrate that CRWN1 and CRWN3 participate in the degradation of ABI5, a positive regulator in the ABA signaling pathway that activates the expression of ABA-responsive genes. In addition, it has been demonstrated that the *crwn1 crwn4* double mutant displays low copper tolerance (Sakamoto et al., 2020). Indeed, CRWN1 is required for the positioning of copper responsive gene clusters on chromosome 5 to the nuclear periphery in order to enhance transcriptional responses under copper induced stress (Sakamoto et al., 2020).

The examples summarized in this chapter demonstrate that the tethering of chromatin to the nuclear periphery *via* NPCs or plant lamin-like proteins can have suppressive as well as enhancing consequences on gene expression depending on the specific locus or stimulus. Given that some *crwn* mutant combinations have partially antagonistic effects on global gene expression patterns (Choi et al., 2019), it will be informative to investigate the functional interplay between the different CRWN proteins and NPCs as well as other components associated with the NE for their roles in regulating gene expression changes and epigenetic transcriptional modifications/memory in response to stimulus perception.

## Concluding Remarks

The work reviewed here emphasizes the plant NPC and its associated transport machinery as an important hub that actively regulates cellular signaling pathways and gene expression as part of developmental programs and in response to environmental factors. Recent work has made significant progress in defining the NPC/NE proteomes, identifying NTR cargo substrates, and in assigning nucleoporin and NTR functions to multiple plant signaling pathways. However, many of the molecular mechanisms underpinning transport-associated and -independent processes in developmental and environmental stress pathways remain to be determined. In particular, open questions to be addressed include: What are the steady-state and stimulus-specific “transportomes” of NTRs and how are NTR cargo binding specificities and transport kinetics across the NPC regulated? Is the nuclear translocation of small molecules, such as plant hormones, diffusion based or actively regulated by binding to transported carrier proteins? What are the effects of positioning particular genes in proximity to the NPC/NE on their expression and epigenetic regulation? How is the recruitment of a certain chromosomal locus regulated, considering that each nucleus contains a multitude of NPCs? Are there tissue/cell-type specific differences in NPC composition and/or post-translational nucleoporin modifications that may account for the involvement in particular response pathways? And finally, is the protein composition of NPCs within a single nucleus identical or variable, and does variability provide sub-populations of NPCs with specific functions? Elucidating these questions will provide valuable insights into the intricate regulation of cellular gene expression and signal transduction pathways by nucleocytoplasmic transport and transport-independent NPC functions in plants.

## Author Contributions

DL and MW conceived the review. DL, PFWR, and MW wrote the manuscript. PFWR prepared the figures and tables. All authors edited and approved the submitted version of the article.

## Conflict of Interest

The authors declare that the research was conducted in the absence of any commercial or financial relationships that could be construed as a potential conflict of interest.

## Publisher’s Note

All claims expressed in this article are solely those of the authors and do not necessarily represent those of their affiliated organizations, or those of the publisher, the editors and the reviewers. Any product that may be evaluated in this article, or claim that may be made by its manufacturer, is not guaranteed or endorsed by the publisher.
